# Engineering *Bacillus licheniformis* for the production of *meso*-2,3-butanediol

**DOI:** 10.1186/s13068-016-0522-1

**Published:** 2016-06-02

**Authors:** Yimin Qiu, Jinyan Zhang, Lu Li, Zhiyou Wen, Christopher T. Nomura, Shuilin Wu, Shouwen Chen

**Affiliations:** Hubei Collaborative Innovation Center for Green Transformation of Bio-Resources, College of Life Sciences, Hubei University, Wuhan, 430062 China; Ministry-of-Education Key Laboratory for Green Preparation and Application of Functional Materials, School of Materials Science and Engineering, Hubei University, Wuhan, 430062 China; State Key Laboratory of Agricultural Microbiology, College of Life Science and Technology, Huazhong Agricultural University, Wuhan, 430070 China; College of Food Science and Technology, Huazhong Agricultural University, Wuhan, 430070 China; Department of Food Science and Human Nutrition, Iowa State University, Ames, IA 50011 USA; Department of Chemistry, The State University of New York College of Environmental Science and Forestry (SUNY ESF), Syracuse, NY 13210 USA

**Keywords:** *Meso*-2, 3-Butanediol, *Bacillus licheniformi*s, *gdh* Gene, Metabolic engineering

## Abstract

**Background:**

2,3-Butanediol (2,3-BD) can be used as a liquid fuel additive to replace petroleum oil, and as an important platform chemical in the pharmaceutical and plastic industries. Microbial production of 2,3-BD by *Bacillus licheniformis* presents potential advantages due to its GRAS status, but previous attempts to use this microorganism as a chassis strain resulted in the production of a mix of *D*-2,3-BD and *meso*-2,3-BD isomers.

**Results:**

The aim of this work was to develop an engineered strain of *B. licheniformis* suited to produce the high titers of the pure *meso*-2,3-BD isomer. Glycerol dehydrogenase (Gdh) was identified as the catalyst for *D*-2,3-BD biosynthesis from its precursor acetoin in *B. licheniformis*. The *gdh* gene was, therefore, deleted from the wild-type strain WX-02 to inhibit the flux of acetoin to *D*-2,3-BD biosynthesis. The *acoR* gene involved in acetoin degradation through AoDH ES was also deleted to provide adequate flux from acetoin towards *meso*-2,3-BD. By re-directing the carbon flux distribution, the double-deletion mutant WX-02Δ*gdh*Δ*acoR* produced 28.2 g/L of *meso*-2,3-BD isomer with >99 % purity. The titer was 50 % higher than that of the wide type. A bench-scale fermentation by the double-deletion mutant was developed to further improve *meso*-2,3-BD production. In a fed-batch fermentation, *meso*-2,3-BD titer reached 98.0 g/L with a purity of >99.0 % and a productivity of 0.94 g/L–h.

**Conclusions:**

This work demonstrates the potential of producing *meso*-2,3-BD with high titer and purity through metabolic engineering of *B. licheniformis*.

**Electronic supplementary material:**

The online version of this article (doi:10.1186/s13068-016-0522-1) contains supplementary material, which is available to authorized users.

## Background

A significant research has been conducted for microbial production of 2,3-butanediol (2,3-BD), as a potential liquid fuel additive for petroleum replacement. As an important platform chemical, 2,3-BD has wide applications in the pharmaceutical and plastic industries [1, 2]. In nature, 2,3-BD exists in three isomeric forms, such as *D*-2,3-BD, *L*-2,3-BD, and *meso*-2,3-BD, with each isomer having its own unique applications. For example, optically active *D*-2,3-BD and *L*-2,3-BD can be used as building blocks in the synthesis of chiral compounds [3], while *meso*-2,3-BD can be used for producing renewable polyesters [4] and enantiomerically pure halohydrins [5], as well as microbial production of 1,3-butadiene [6], 2-butanol, and butanone [7, 8]. Natural organisms usually produce 2,3-BD in the form of a mix of two of three isomers, with the ratio of products dependent on the strains and fermentation conditions used [9]. To reduce the cost of purifying a specific 2,3-BD isomer from fermentation broth, it is desirable for the microbial strain to produce the specific target isomer without the synthesis of the other forms of 2,3-BD.

Genetic engineering has been used to produce specific 2,3-BD isomers with high purity. For example, *D*-2,3-BD was produced with a purity >97.5 % through an engineered *Enterobacter cloacae* strain to titers of 152.0 g/L [10]. Engineered *Escherichia coli* has also been used to produce *L*-2,3-BD from diacetyl with a purity >99 % [3]. A high purity (~98 %) of *meso*-2,3-BD was also reported through a recombinant *E. coli* strain [11]. However, the titer (17.7 g/L) and yield (0.18 g/g glucose) of *meso*-2,3-BD produced from this system were very low [11], making economic industrial application difficult.

**Fig. 1:**
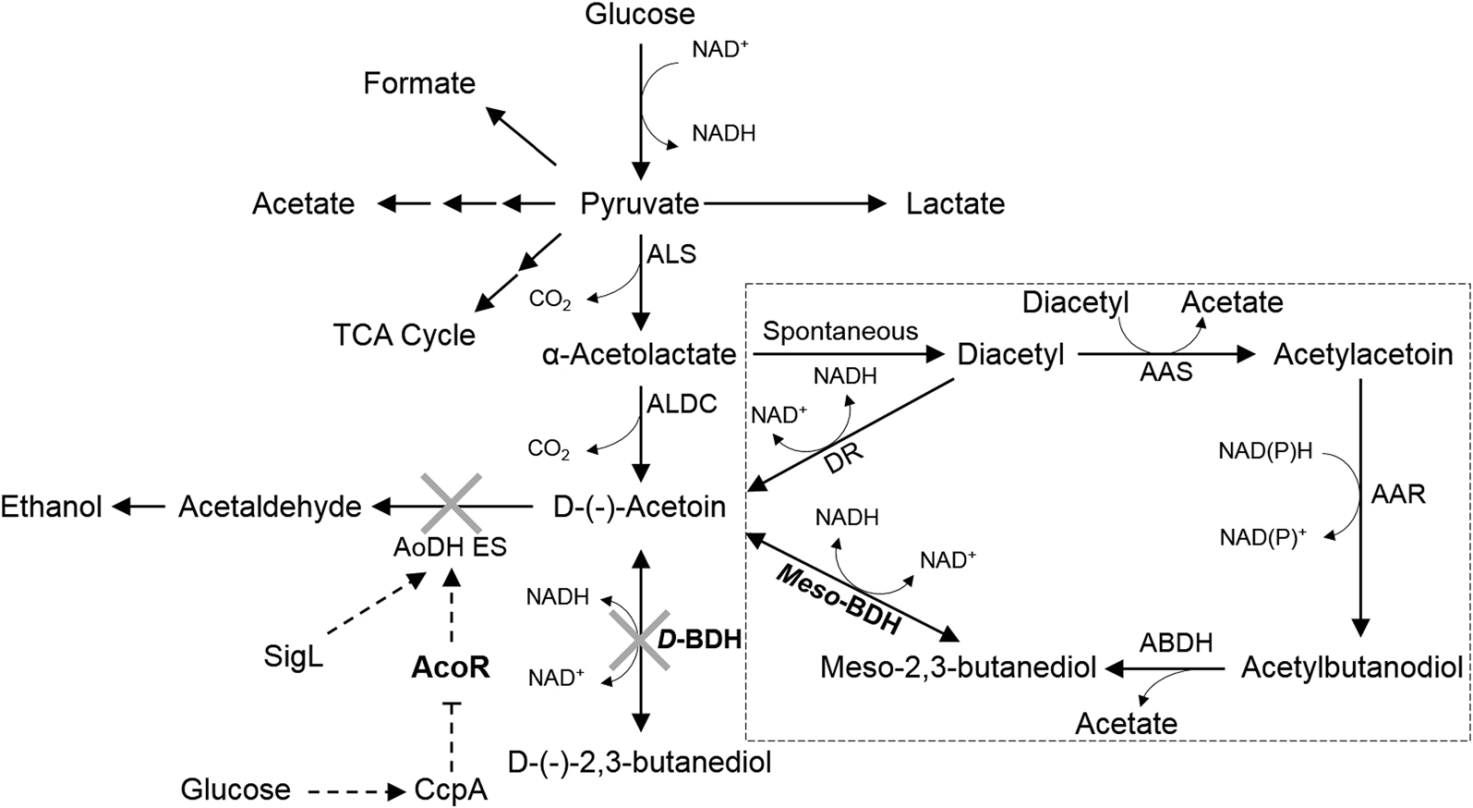
Metabolic pathway of 2,3-butanediol synthesis in *B. licheniformis* WX-02 (modified based on [9, 24, 41, 44]). *ALS* α-acetolactate synthase, *ALDC* α-acetolactate decarboxylase, *BDH* 2,3-butanediol dehydrogenase, *AAS* acetylacetoin synthase, *AAR* acetylacetoin reductase, *DR* diacetyl reductase, *AoDH ES* acetoin dehydrogenase enzyme system. The *solid arrows* indicate the reactions; the *dashed arrows* indicate the regulatory events; the *dashed square* marks the hypothetical 2,3-butandiol cycle; the *crosses* indicate the disruption of the target pathways in this work

Many microorganisms are capable of producing 2,3-BD, such as *Enterobacter*, *Klebsiella*, *Serratia*, and *Bacillus.* Among them, *Bacillus licheniformis* is a promising chassis strain for 2,3-BD production. It is generally regarded as safe (GRAS) and used as an expression platform for producing enzymes, amino acids, antibiotics, and other secondary metabolites [12]. Natural *B. licheniformis* strains usually produce a mixture of *D*-2,3-BD and *meso*-2,3-BD isomers. Figure 1 shows the proposed 2,3-BD biosynthesis pathway in natural *B. licheniformis*. The precursor of 2,3-BD isomers is *D*-(−) acetoin (*D*-AC), which is reduced to *D*-2,3-BD and *meso*-2,3-BD through two 2,3-butanediol dehydrogenases (BDHs), *D*-BDH and *meso*-BDH, respectively [9]. The conversion between 2,3-BD and *D*-AC is reversible and coupled with NAD^+^/NADH conversion. The enzyme *meso*-BDH was confirmed in our earlier study [13], while *D*-BDH is a putative enzyme and had not been confirmed to catalyze the conversion of *D*-AC to *D*-2,3-BD. Oxygen supply is a critical factor for AC and 2,3-BD inter-conversion. High dissolved oxygen (DO) levels lead to AC synthesis, while low DO levels result in 2,3-BD synthesis [14–16]. In addition to serving as a precursor for 2,3-BD, *D*-AC also serves as a carbon source by *B. licheniformis* when glucose is depleted from the medium [17]. This metabolism of AC is mediated by the acetoin dehydrogenase enzyme system (AoDH ES), which is encoded by the *acoABCL* operon. Transcription of *aco* operon is activated by AcoR and SigL (sigma factor), and inhibited by CcpA-based catabolite repression. It had been reported that blocking AC catabolism resulted in an accumulation of this compound, which contributed to 2,3-BD synthesis [18].

The metabolic pathway in Fig. 1 provides guidance for engineering *B. licheniformis* for producing the targeted 2,3-BD isomer with high purity. Qi et al. [13] have engineered a strain to produce the pure *D*-2,3-BD isomer to titers of 30.8 g/L and a purity >95.4 %. However, *meso*-2,3-BD production by this strain had not been attempted. The aim of this work was to develop a metabolic engineering approach to produce pure *meso*-2,3-BD isomer by altering the carbon flux by blocking the *D*-2,3-BD biosynthesis and acetoin degradation pathways. In addition, the enzyme that catalyzes the conversion of *D*-AC to *D*-2,3-BD in *B. licheniformis* was also elucidated in this study.

## Results

### Identification of the enzyme catalyzing *D*-AC to *D*-2,3-BD in *B. licheniformis*

To produce *meso*-2,3-BD with high optical purity in *B. licheniformis*, we proposed to block the synthesis of *D*-2,3-BD by knocking out the gene encoding the enzyme catalyzing the conversion of *D*-AC to *D*-2,3-BD, so that the carbon flux from *D*-AC would be diverted into *meso*-2,3-BD (Fig. 1). It was hypothesized that the reversible conversion of *D*-AC to *D*-2,3-BD was catalyzed by a putative *D*-2,3-butanediol dehydrogenase (*D*-BDH) enzyme (Fig. 1). Although gene *bdhA* encoding the *D*-BDH enzyme had been identified in *B. subtilis* [12]), the analysis of the *B. licheniformis* genome did not reveal the existence of any homologs to *bdhA*. Alternatively, researchers have reported that the glycerol dehydrogenase (GDH) in *B. licheniformis* is capable of catalyzing the in vitro conversion of *D*-AC to *D*-2,3-BD [19]. Similar activities for GDHs (DhaD and GldA) were also observed in *K. pneumonia* [20]. Based on these results, the *gdh* gene in *B. licheniformis* was studied for its role in *D*-2,3-BD biosynthesis.

A 1104-bp putative GDH encoding gene (*gdh*) was identified in *B. licheniformis* WX-02 [21]. The deduced amino acid sequence of the protein (WX-02 GDH) was compared with GDHs reported in other strains via sequence alignments through ClustalW. Comparative analysis revealed that WX-02 GDH had nearly all the conserved residues of the iron-dependent alcohol dehydrogenase (Fe-ADH), including three glycine residues that are predicted to interact with the NAD(H) cofactor and three histidine residues that coordinate an iron cofactor. This suggests that the WX-02 GDH is a member of the Fe-ADH superfamily. In addition, a strong similarity (>48 %) was found between WX-02 GDH and GDHs, exhibiting *D*-BDH activity from other organisms, such as *Serratia marcescens* [22], *Klebsiella pneumoniae* (DhaD, KJ206474.1), *Citrobacter freundii* (DhaD, P45511.1), *Klebsiella oxytoca* (DhaD, YP_005016612), and *B. licheniformis* 10-1-A (GldA, AHW84748.1).

**Fig. 2:**
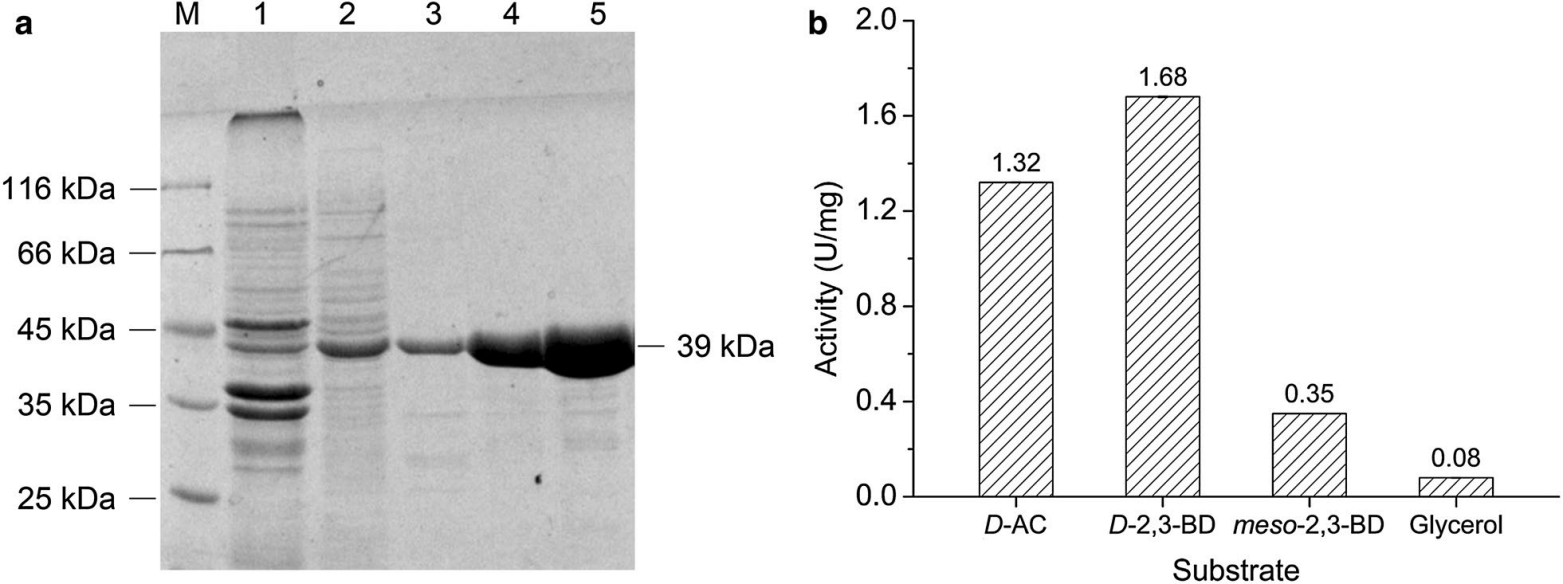
Expression and catalytic activity of GDH from *B. licheniformis* WX-02 (WX-02 GDH). **a** SDS-PAGE of the expression and purification of WX-02 GDH. The recombinant *E. coli* BL21(DE3)/pET-*gdh* was grown to the appropriate density and induced with IPTG for the production of the GDH protein. The total-cell extracts from the induced cells were separated into soluble and insoluble fractions. Proteins in the soluble fractions were purified by the Ni–NTA purification kits, and the GDH protein was purified. *Lane 1* sediment of cell extracts; *lane 2* supernatant of cell extracts; *lane 3* purified GDH protein in 50-fold dilution; *lane 4* purified GDH protein in tenfold dilution; *lane 5* purified GDH protein. **b** Activities of purified WX-02 GDH on different substrates with corresponding coenzymes

As shown in Fig. 2a, the His-tagged recombinant GDH protein was efficiently expressed in soluble form after IPTG induction. The purified GDH protein was observed as a single band on SDS-PAGE with an approximate molecular weight consistent with that predicted from its amino acid sequence (39.5 kDa). The activity of purified GDH from WX-02 was further investigated for its catalytic activities on various substrates with coenzymes. Figure 2b showed that WX-02 GDH accepted *D*-AC, *D*-2,3-BD, *meso*-2,3-BD, and glycerol as substrates. However, this enzyme exhibited the highest activity towards *D*-2,3-BD among various substrates. Its catalytic activity on glycerol was only around 5 % of that for *D*-2,3-BD. The preference on *D*-2,3-BD as the substrate by GDH was also reported in *Hansenula polymorpha* [23]. These results indicated that the GDH from *B. licheniformis* WX-02 possessed a substrate specific catalytic activity towards *D*-2,3-BD. Based on these observations, we concluded that the GDH enzyme was the best candidate enzyme for catalyzing *D*-2,3-BD synthesis in *B. licheniformis* WX-02. Consequently, we decided to delete the *gdh* gene, so the conversion of *D*-AC to 2,3-BD isomers could be diverted solely towards *meso*-2,3-BD production.

### Deletion of *gdh* gene for production of *meso*-2,3-BD with high purity

**Fig. 3:**
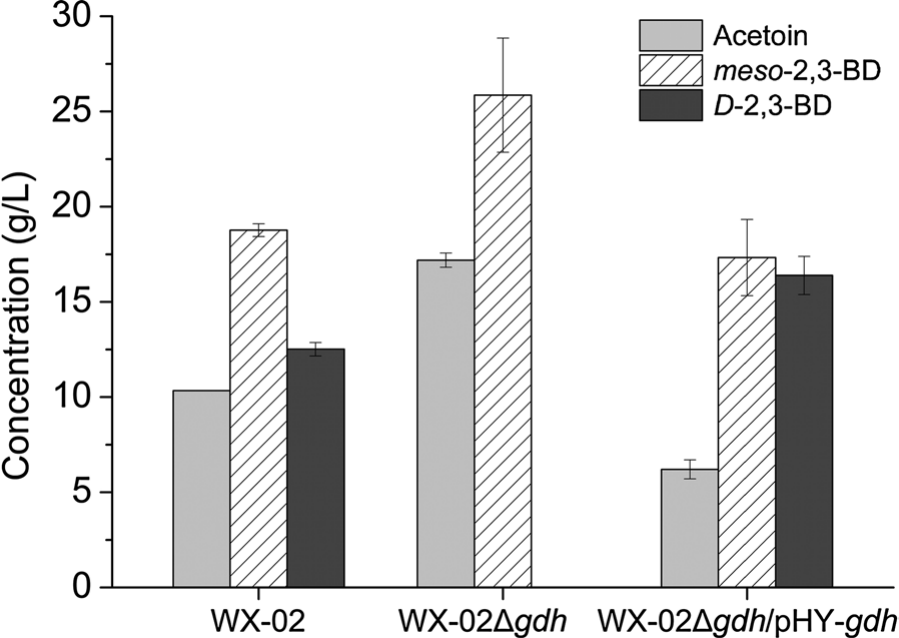
Production of acetoin and 2,3-BD isomers by *B. licheniformis* WX-02 and the *gdh* mutant strains. *WX-02* wild-type strain, *WX-02*Δ*gdh* the mutant strain with *gdh* gene knocked out from the genome of WX-02, *WX-02*Δ*gdh/PHY-gdh* the mutant strain with *gdh* gene complemented to the knock-out strain WX-02Δ*gdh.* The cells were grown under shake-flask culture conditions for 24 h. Data are means of three replicates, and *error bars* show standard deviations

To investigate the role of GDH in the biosynthesis of 2,3-BD isomers, we constructed WX-02Δ*gdh*, a *gdh*-deficient strain of *B. licheniformis* WX-02. As shown in Fig. 3, WX-02 produced both *meso*-2,3-BD and *D*-2,3-BD, while no *D*-2,3-BD was produced by WX-02Δ*gdh*. Complementation of *gdh* in WX-02Δ*gdh* (strain WX-02Δgdh/pHY-gdh) restored its ability to produce *D*-2,3-BD. Figure 3 also showed dramatic differences in the production of 2,3-BD and *D*-AC between the wild-type and the engineered strain. Compared to WX-02, production of *meso*-2,3-BD and *D*-AC increased by 66.3 and 37.8 %, respectively, in WX-02Δ*gdh*. The results indicate that the deletion of *gdh* gene from the wild-type strain eliminates the synthesis of *D*-2,3-BD, leading to an accumulation of its precursor *D*-AC, which in turn promoted the conversion of *D*-AC to *meso*-2,3-BD. *Meso*-2,3-BD was the only 2,3-BD isomer detected in the medium, resulting in a product of high purity. It was also found that the mutant WX-02Δ*gdh*/pHY-*gdh* not only restored the production of *D*-2,3-BD but produced an even higher titer of total 2,3-BD isomers (*D*-2,3-BD and *meso*-2,3-BD) compared to that of wild-type. Correspondingly, the *D*-AC produced by the complementation strain was lower than the wild-type WX-02. The distribution profile of these metabolites indicates that the high expression of *gdh* gene is likely a result of the strong promoter P43 and multiple copies of pHY300PLK vector.

### Deletion of *acoR* gene for accumulation of acetoin

**Fig. 4:**
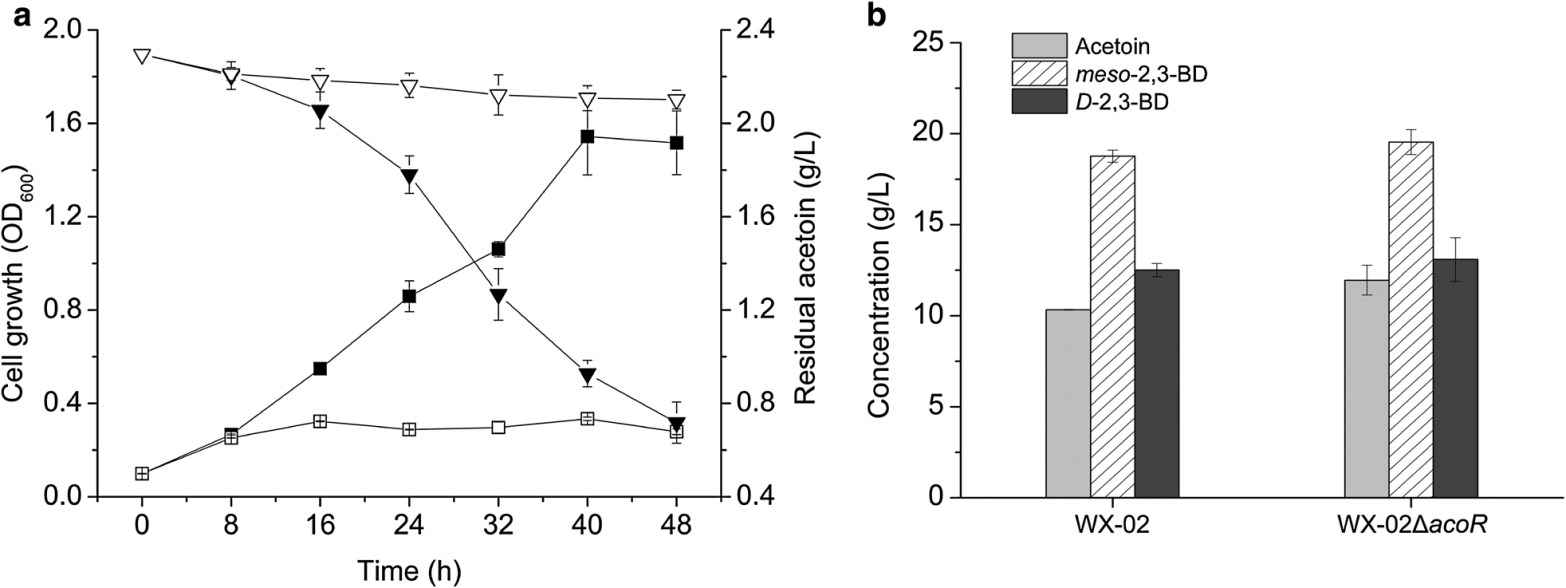
The effect of acoR knock-out on acetoin metabolism of *B. licheniformis*. **a** Time course of the growth (*squares*) and acetoin consumption (*triangles*) by the *acoR*-disrupted strain (WX-02Δ*acoR*) (*open symbols*) and wild-type strain (WX-02) (*solid symbols*) in medium with acetoin (2.3 g/L) as the sole carbon source. **b** The accumulation of acetoin and 2,3-BD isomers by the mutant strain (WX-02Δ*acoR*) and wild-type strain (WX-02) (at 24-h of culture) in medium with glucose (120 g/L) as carbon source. The cells were grown under the shake-flask culture with minerals in 2,3-BD production medium were used for each cases. Data are means of three replicates, and *error bars* show standard deviations

In addition to serving as the precursor for 2,3-BD synthesis, acetoin has also been reported to be used as a carbon source by *B. licheniformis*. This is regulated by AoDH ES (Fig. 1) when glucose was depleted [17, 24]. Acetoin dissimilation would reduce its availability for *meso*-2,3-BD synthesis resulting in an adverse effect on 2,3-BD production. In this work, the *acoR* gene encoding transcriptional activator of AoDH ES was disrupted to block the degradation of acetoin. Two separate experiments were then performed to confirm the effect of the *acoR* knock-out on acetoin metabolism. First, the *acoR*-disrupted strain WX-02Δ*acoR* was grown in the medium containing acetoin as the sole carbon source. The wild-type strain WX-02 was also grown in this medium as a control. As shown in Fig. 4a, the mutant WX-02Δ*acoR* had a very poor growth compared to that of the wild-type WX-02. The poor growth caused by the acetoin utilization deficiency was also observed in *B. subtilis* and *B. licheniformis* strains with inactivated *acoABCL* operons [17, 25, 26]. The results of the acetoin consumption study presented in Fig. 4a also indicated the inability of utilizing acetoin by the *acoR*-disrupted mutant. Second, WX-02Δ*acoR* and WX-02 were grown in 2,3-BD production medium containing glucose as the carbon source. As shown in Fig. 4b, the mutant WX-02ΔacoR produced 15.7 and 4.3 % more *D*-AC and total 2,3-BD isomers than the wild-type WX-02, respectively. The results clearly demonstrate the role of *acoR* in *D*-AC metabolism, and the deficiency of *acoR* contributed to *D*-AC accumulation, thus favoring 2,3-BD production.

### Double deletion of *gdh* and *acoR* for producing *meso*-2,3-BD with high purity and titer

The above results demonstrated that single deletions of *gdh* or *acoR* gene were beneficial for the production of *meso*-2,3-BD production in terms of both titer and purity. Compared to the wild-type strain WX-02, a *gdh* gene deletion resulted in the sole production of *meso*-2,3-BD, while deletion of the *acoR* gene led to an accumulation of *D*-AC, the precursor for 2,3-BD synthesis. To maximize *meso*-2,3-BD biosynthesis, we engineered a strain with both *gdh* and *acoR* genes deleted. The metabolites produced by the double-gene-deletion mutant WX-02Δ*gdh*Δ*acoR* were compared to those produced by the wild-type and the single-gene deficient strains. As shown in Table 1, the mutant WX-02Δ*gdh*Δ*acoR* produced 28.2 g/L of *meso*-2,3-BD, 50.3 % higher than that of the wild-type WX-02. The *meso*-2,3-BD yield and productivity of the double-deletion strain were also significantly higher with respect to the wild-type. Disruption of the *gdh* and *acoR* genes also affected the synthesis of other metabolites, such as lactic acid, acetic acid, and ethanol. Table 1 shows that the mutant strains, particularly WX-02Δ*gdh*Δ*acoR*, produced lower amounts of these by-products, indicating the benefit of metabolic engineering of *B. licheniformis* for *meso*-2,3-BD production.

**Table 1:** Production of various metabolites by different *B. licheniformis* strains

*B. licheniformis* strain	Biomass (OD_600_)	Metabolites (g/L)						*meso*-2,3-BD yield^a^ (%)	*meso*-2,3-BD productivity^b^ (g/L–h)
		*meso*-2,3-BD	*D*-2,3-BD	Acetoin	Lactic acid	Acetic acid	Ethanol		
WX-02	29.60 ± 0.82	18.77 ± 0.33	12.51 ± 0.36	10.33 ± 0.02	2.21 ± 0.12	0.46 ± 0.06	0.73 ± 0.12	15.64	0.78
WX-02Δ*gdh*	28.80 ± 0.24	25.86 ± 3.00	ND	17.19 ± 0.37	2.13 ± 0.02	0.21 ± 0.01	0.54 ± 0.03	21.55	1.08
WX-02Δ*acoR*	28.05 ± 0.88	19.54 ± 0.69	13.10 ± 1.19	11.96 ± 0.91	1.94 ± 0.07	0.25 ± 0.07	0.62 ± 0.08	16.29	0.81
WX-02Δ*gdh*Δ*acoR*	30.03 ± 0.69	28.22 ± 0.82	ND	17.16 ± 0.66	1.63 ± 0.17	0.11 ± 0.04	0.51 ± 0.14	23.52	1.18

Strains were grown in 250-mL flasks containing 50-mL medium and incubated in a rotary shaker with 180 rpm at 37 °C for 24 h. The initial glucose concentration was 120 g/L, which was completely consumed by the cells at the end of culture; Data are presented as mean ± SDs of three replicates

^a^The yield was calculated as the weight ratio of *meso*-2,3-BD produced to glucose consumed

^b^The productivity was calculated by dividing maximum *meso*-2,3-BD concentration to the time when the cells were harvested

### Optimization of *meso*-2,3-BD production by the mutant WX-02Δ*gdh*Δ*acoR*

To this point, the previously described results demonstrated the capability of the mutant WX-02Δ*gdh*Δ*acoR* to produce *meso*-2,3-BD with a high purity and titer. A bench-scale fermentation was further developed in both batch and fed-batch modes to explore the potential of commercial production of *meso*-2,3-BD by this double-deletion mutant.

**Fig. 5:**
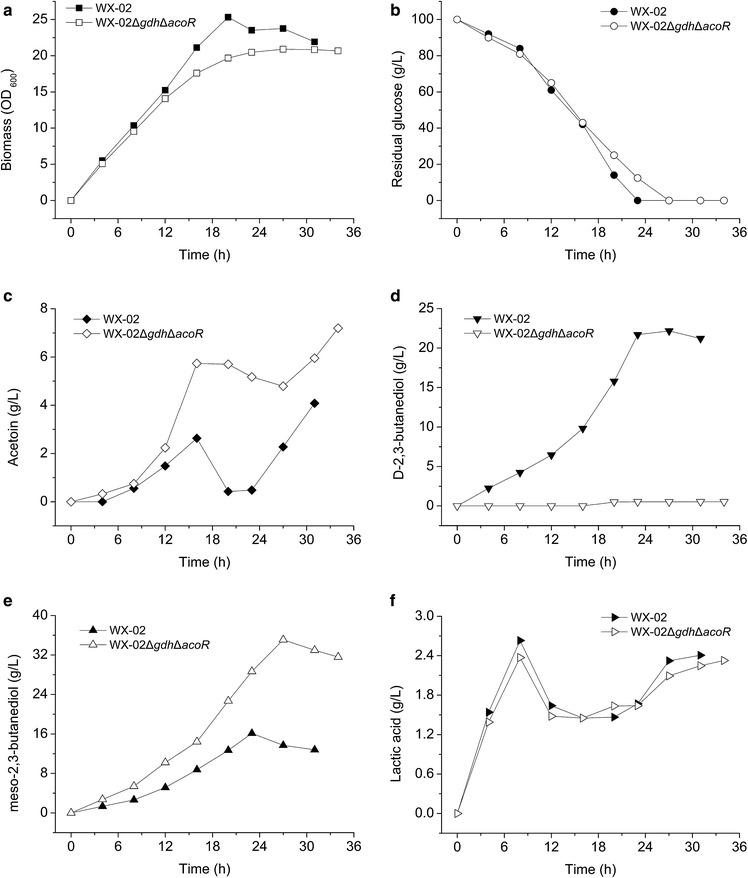
Batch fermentation profile of *B. licheniformis* strains WX-02 and WX-02Δ*gdh*Δ*acoR* in a bench top (5-L) fermenter. **a** Biomass density; **b** residual glucose; **c** acetoin production; **d** *D*-2,3-BD production; **e** *meso*-2,3-BD production; **f** lactic acid production

Figure 5 shows the batch fermentation profiles. The growth (Fig. 5a) and glucose consumption (Fig. 5b) of the mutant WX-02Δ*gdh*Δ*acoR* were similar to the wild-type WX-02 strain. The WX-02Δ*gdh*Δ*acoR* strain accumulated higher acetoin (Fig. 5c), but negligible *D*-2,3-BD isomer (Fig. 5d) compared to the wild-type. The *meso*-2,3-BD isomer produced from WX-02Δ*gdh*Δ*acoR* strain was double that of the wild-type strain (Fig. 5e). The yield of *meso*-2,3-BD from glucose, *Y*_*meso*-2,3-BD/glucose_, was 0.35 g/g for WX-02Δ*gdh*Δ*acoR*, also much higher than wild-type (0.16 g/g). Finally, lactic acid was still produced as the main by-product (Fig. 5f), while acetic acid and ethanol were less than 1.0 g/L throughout the fermentation process (data not shown). It should be noted that the lactic acid produced by the mutant WX-02Δ*gdh*Δ*acoR* was similar to that produced by the wide type strain under fermenter culture conditions (Fig. 5f). This trend is different from that in flask culture (Table 1), where WX-02Δ*gdh*Δ*acoR* produced less lactic acid than WX-02. The reason may be due to the different operation conditions between the flask culture and the fermenter culture.

Compared to the shake-flask culture results (Table 1), both the wild-type and WX-02Δ*gdh*Δ*acoR* strains produced more *meso*-2,3-BD with less accumulation of acetoin in the fermenter culture. This was due to the use of the two-stage agitation/aeration, and thus the controlled dissolved oxygen (DO) levels, during the fermenter operation. It has been reported that DO levels play a crucial role in the reversible conversion of acetoin to 2,3-BD [14–16, 19]. In this work, the relatively high DO level generated by high aeration and agitation during the initial fermentation stage produced an increased amount of acetoin, resulting in the favorable conversion to *meso*-2,3-BD during the second stage when DO was intentionally reduced to a lower level.

**Fig. 6:**
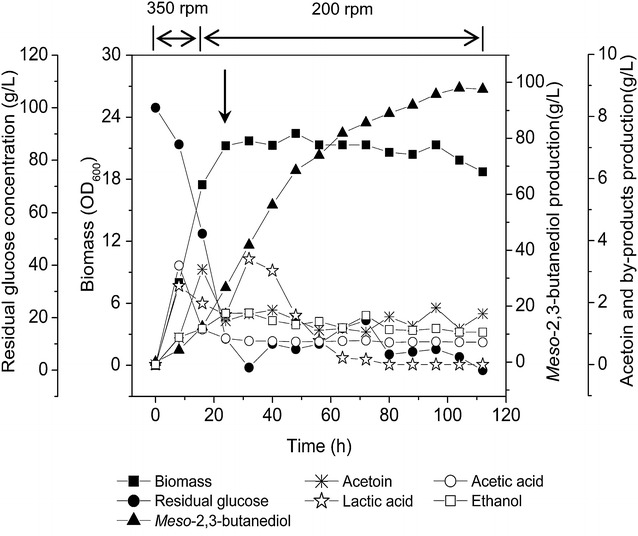
Fed-batch fermentation profiles of *B. licheniformis* mutant WX-02Δ*gdh*Δ*acoR* in a *bench top* (5-L) fermenter with pH control. The tank was stirred at 350 rpm with 3-L/min aeration for first 16 h, and then decreased to 200 rpm and 1.5 mL/min for the remaining period. Glucose was fed to the medium from 24–70 h to maintain the residual glucose concentration between 10–20 g/L. *Filled square* Biomass; *filled circle* residual glucose; *filled triangle* *meso*-2,3-BD; ❊, Acetoin; ☆, Lactic acid; *circle* acetic acid; *square* ethanol. The *arrow* indicates the start of feeding the reactor with a concentrated glucose solution (650 g/L)

A fed-batch fermentation using WX-02Δ*gdh*Δ*acoR* was further developed to improve the *meso*-2,3-BD isomer titer. As shown in Fig. 6, glucose was maintained at 10-20 g/L throughout the entire culture period through periodic feedings. *Meso*-2,3-BD titer reached up to 98.0 g/L with a yield *Y*_*meso*-2,3-BD/glucose_ of 0.40 g/g and productivity of 0.94 g/L–h, which was the highest *meso*-2,3-BD yield reported in *Bacillus* species (Table 2).

**Table 2:** 2,3-Butanediol (2,3-BD) production by native or engineered *Bacillus* strains

Strain	Substrate	2,3-BD isomer	Method	Concentration (g/L)	Yield (g/g)	Productivity (g/L–h)	References
*Bacillus subtilis*							
AJ 1992 (WT)	Glucose	Mixture	Batch	2.5	0.38	0.33	[36]
RB03^a^	Glucose	Mixture	Batch	6.1	0.34	0.41	[37]
*Bacillus amyloliquefaciens*							
F (WT)	Glucose	Mixture	Batch	33.0	0.33	–	[9]
B10-127 (WT)	Glucose	Mixture	Shake-flask	55.8	0.40	0.80	[38]
			Fed-batch	92.3	0.15	0.96	
GAR^b^	Crude glycerol	Mixture	Fed-batch	102.3	0.44	1.16	[39]
*Bacillus licheniformis*							
Wild-type strain	Glucose	Mixture	Static flask	8.7	0.47	0.12	[40]
BL5^c^	Glucose	*D*-2,3-BD	Batch	12.2	0.45	2.65	[41]
WX-02Δ*budC*	Glucose	*D*-2,3-BD	Shake-flask	30.8	0.27	1.28	[13]
X10 (WT)	Concentrated corn stover hydrolysate	Mixture	Fed-batch	74.0	0.47	2.06	[42]
DSM 8785 (WT)	Glucose	*D*-2,3-BD	Batch	72.6	0.42	0.86	[43]
			Fed-batch	144.7	0.40	1.14	
			Immobilized	118.3	0.41	1.14	
WX-02Δ*gdh*Δ*acoR*	Glucose	*Meso*-2,3-BD	Shake-flask	28.2	0.24	1.18	This work
			Batch	35.1	0.35	1.30	This work
			Fed-batch	98.0	0.40	0.94	This work

^a^ *B. subtilis* 168 containing pAL10abA (P_*alsSD*_-*bdhA*)

^b^ *B. amyloliquefaciens* B10-127 with pMA5-*acr*-HapII-*dha*D-P_bdhA_-*alsR*

^c^ *B. licheniformis* BL1Δ*ldh*

## Discussion

Of the 2,3-BD isomers, *meso*-2,3-BD is an excellent platform chemical with numerous applications [4–8]. The ability to microbially produce *meso*-2,3-BD in a pure form is crucial for improving commercial viability. Examples of 2,3-BD production using native microorganisms, such as *Klebsiella* and *Enterobacter* spp., produced a mix of *meso*-2,3-BD and *L*-2,3-BD [22], whereas *Bacillus* spp. produced a *meso*-2,3-BD and *D*-2,3-BD mixture [19]. The resulting mixture of 2,3-BD isomers is difficult to purify with high downstream costs.

The wild-type strain *B. licheniformis* WX-02 produces a mix of *D*-2,3-BD and *meso*-2,3-BD [13]. It has been confirmed that different 2,3-BD isomers arise from the same precursor *D*-AC, and the enzyme catalyzing the reduction of *D*-AC to *meso*-2,3-BD (*meso*-2,3-butanediol dehydrogenases) has been identified in *B.licheniformis* WX-02 [13]. However, bioinformatics studies revealed that the putative gene *D*-2,3-BDH (D-2,3-butanediol dehydrogenases) encoding the enzyme catalyzing the reduction of *D*-AC to *D*-2,3-BD was not present in the genome of *B. licheniformis* WX-02. This trait is different from species, such as *Paenibacillus polymyxa* ZJ-9, *S. cerevisiae* S288c, and *Clostridium beijerinckii* NRRLB593, where *D*-2,3-BDHs were identified as the catalysts.

On the other hand, glycerol dehydrogenases (GDH) have been reported to play an important role in 2,3-BD synthesis in *K. pneumoniae* and *S. marcescens* [27, 28]. In this work, the *B.licheniformis* WX-02 GDH was identified. The primary structure of the enzyme is very similar to amino acid sequences of GDHs from other microbes with the *D*-BDH function (Additional file 1: Figure S1). This led us to explore the function of GDH as a catalyst for the conversion of *D*-AC to *D*-2,3-BD in *B.licheniformis* WX-02. The purified WX-02 GDH exhibited extremely high activity for *D*-2,3-BD (Fig. 2). Characterization and complementation of the *gdh*-deficient mutant *B.licheniformis* WX-02Δ*gdh* further confirmed the catalytic role of WX-02 GDH in the conversion of *D*-AC to *D*-2,3-BD. A similar substrate range was observed for the GDH from other strains, such as *Serratia*, *Klebsiella* and *Hansenula* species [20, 23, 28].

Although phylogenetic analysis showed that the enzymes GDH and *D*-BDH belonged to different branches (Additional file 2: Figure S2) [29], based on homology; these two enzymes belong to the medium-chain dehydrogenase/reductase (MDR) family [20]. Other GDHs, such as *O. angusta* GDH, also show homology with *D*-BDHs (Additional file 2: Figure S2). The catalytic function of GDH in *B.licheniformis* WX-02 for converting *D*-AC to *D*-2,3-BD is likely due to the evolution of the enzyme [20].

The production of optically pure 2,3-BD isomers as intermediates for the pharmaceutical industry is highly desirable [20]. In this work, two metabolic engineering methods were used to produce high levels of pure *meso*-2,3-BD. First, the *gdh* gene was knocked out to block *D*-2,3-BD formation (Fig. 1). The engineered *B.licheniformis* WX-02Δ*gdh* strain significantly increased *meso*-2,3-BD production and completely eliminated the production of the competing product *D*-2,3-BD. Second, the key transcriptional regulator AcoR in the acetoin degradation pathway was disrupted through the deletion of *acoR* gene. This practice proved effective in *D*-AC accumulation in WX-02Δ*acoR*; therefore, the flux from *D*-AC to 2,3-BD could be increased. Based on these two independent developments, a double-deletion *gdh*-*acoR-B.licheniformis* WX-02 strain was developed. The resulting strain, WX-02Δ*gdh*Δ*acoR*, produced increased *meso*-2,3-BD titers (28.2 g/L) in shake-flask cultures, ~1.5-fold higher than the titers observed for the wild-type (Table 1). The high production of *meso*-2,3-BD was also accompanied by less formation of by-products, such as lactic acid, acetic acid, and ethanol (Table 1). More importantly, *meso*-2,3-BD was the only 2,3-BD isomer produced by the double-deletion mutant (Fig. 5; Table 1).

A two-stage aeration/agitation control method had previously proven as an efficient way to enhance the 2,3-BD isomers production. This was accomplished by controlling the dissolved oxygen (DO) levels during the fermentation. The high DO levels generated by relatively high agitation speeds favored acetoin production [14, 15, 30]. Conversely, a lower DO level was generated by decreasing the agitation speed which favored the conversion of AC to 2,3-BD [16]. Here, we used a similar strategy to improve the production of *meso*-2,3-BD isomer by WX-02Δ*gdh*Δ*acoR* in a bench-scale fermentation system. The use of fed-batch fermentation resulted in *meso*-2,3-BD titers of 98.0 g/L and a productivity of 0.94 g/L–h. As a comparison, other researchers have reported titers of 73.8 g/L of *meso*-2,3-BD with 95.5 % purity using a recombinant *E. coli* [2]. Therefore, the *meso*-2,3-BD titers obtained in this work, together with its high purity (>99.0 %), indicate that these are the highest levels of microbial *meso*-2,3-BD produced so far. Table 2 further summarizes the production of various 2,3-BD isomers by *Bacillus* strains. The titer, yield, and purity of *meso*-2,3-BD isomer obtained from this study are among the highest reported in the literature, indicating that the great potential of commercially producing this important 2,3-BD isomer using the engineered *B*. *licheniformis* strains reported in this work.

## Conclusions

The present study reveals the role of GDH in *D*-2,3-BD biosynthesis in *B. licheniformis*. Engineered *B. licheniformis* with deletions of the *gdh* and *acoR* genes was able to produce the *meso*-2,3-BD isomer with high titers. The fed-batch fermentation developed in this work resulted in highest titer (98.0 g/L) ever reported for *meso*-2,3-BD. This work demonstrates the potential of using engineered *B. licheniformis*, as a chassis for the industrial production of pure *meso*-2,3-BD.

## Methods

### Strains and plasmids

The strains and plasmids used in this study were listed in Table 3. *Escherichia coli* DH5α and BL21(DE3) were used as hosts for cloning and gene expression, respectively. All *B. licheniformis* strains were derived from the wild-type WX-02 (CCTCC M208065) [31]. The vectors pET-28a(+) and pHY300PLK were used for protein expression in *E. coli* BL21(DE3) and *B. licheniformis* WX-02, respectively. The T2(2)-ori vector was a shuttle plasmid for gene deletions in *B. licheniformis*, with a temperature-sensitive replicon from *B. subtilis* to promote single-crossover events [32].

**Table 3:** Strains and plasmids used in this study

Strain or plasmid	Relevant genotype/description	Source
Strains		
*B. licheniformis*		
WX-02	CCTCC M208065, wild-type strain	Laboratory stock
WX-02Δ*gdh*	*gdh*-knock-out mutant of WX-02	This work
WX-02Δ*gdh*/pHY-*gdh*	recombinant strain of WX-02Δ*gdh*, harboring pHY-*gdh*, Tc^r^	This work
WX-02Δ*acoR*	*acoR* knock-out mutant of WX-02	This work
WX-02Δ*gdh*Δ*acoR*	*gdh* and *acoR* double-deletion mutant of WX-02	This work
*E. coli*		
DH5α	F^–^ Φ80d/*lac*ZΔM15, Δ (*lacZYA*-*argF*) U169, *recA*1, *endA*1, *hsdR*17 (*r*_K_^–^, *m*_K_^+^), *phoA*, *supE*44, λ-, *thi*-1, *gyrA*96, *relA*1	Laboratory stock
BL21(DE3)	F^–^, *omp*T, *hsdS*_*B*_ (*r*_*B*_^−^ *m*_*B*_^−^), *gal*, *dcm* (DE3)	Laboratory stock
BL21(DE3)/pET-*gdh*	*E. coli* BL21(DE3) harboring pET-*gdh*, Kan^r^	This work
Plasmids		
pET-28a(+)	*E. coli* expressing vector, Kan^r^	Laboratory stock
pET-*gdh*	pET-28a(+) carrying *gdh* gene	This work
pHY300PLK	*E. coli*-*Bacillus* shuttle vector, Amp^r^ in *E. coli,* Tc^r^ in both *E. coli* and *B. subtilis*	Laboratory stock
pHY-*gdh*	pHY300PLK carrying *gdh* gene	This work
T2(2)-ori	*E. coli*-*B. licheniformis* shuttle vector, Ori_pUC_/Ori_ts_, Kan^r^	Laboratory stock
T2Δ*gdh*	T2(2)-ori derivative containing homologous arms for *gdh*-knock-out	This work
T2Δ*acoR*	T2(2)-ori derivative containing homologous arms for *acoR* knock-out	This work

*Tc* ^*r*^ tetracycline resistance, *Kan* ^*r*^ kanamycin resistance gene, *Amp* ^*r*^ ampicillin resistance gene, *Ori*_*ts*_ thermosensitive replication origin. For recombinant mutants, 100-μg/mL Ampicillin, 20-μg/mL kanamycin, or 20-µg/mL tetracycline were used when necessary

### Medium preparation and culture conditions

*Escherichia coli* and *B. licheniformis* seed cultures were performed in 250-mL flasks containing 50-mL LB medium at 37 °C on a rotary shaker (180 rpm). For 2,3-BD production, the seed culture with a OD_600_ at 4.0 was inoculated (1 %, v/v) into either 250-mL flasks containing 50-mL medium or into a 5-L stir tank fermenter (Guo Qiang Bioengineering Equipment Company, Ltd. China) with a working volume of 3 L. The medium consisted of (per liter): glucose 120 g, corn steep liquor 33 g, (NH_4_)_2_SO_4_ 9.0 g, K_2_HPO_4_ 1.0 g, MgSO_4_ 1.5 g, NaCl 0.5 g, ZnCl_2_ 0.12 g, FeCl_3_ 0.001 g, and MnSO_4_ 0.001 g, with an initial pH adjusted to 7.0 before autoclaving at 121 °C for 15 min [13]. Fermentations were carried out at 37 °C. The batch and fed-batch fermentation were operated under optimized conditions as follows: the pH was maintained at 6.0 with 6-M HCl or 7.5-M ammonia hydroxide; aeration, and agitation were, respectively, controlled at 3 L/min and 350 rpm for the first 16 h, and 1.5 L/min, and 200 rpm for the rest of fermentation. In the fed-batch operation, in addition to the above conditions used, a concentrated glucose solution (650 g/L) was added to the fermenter at a rate of 15 mL/h from 24–70 h to maintain the glucose concentration in the medium at a level of 10–20 g/L. As a result, the total glucose added to the fermenter was equivalent to 150 g/L.

### Construction of the gene deletion mutants of *B. licheniformis*

The mutants with single-gene deletions of *gdh* or *acoR* and a mutant with both *gdh* and *acoR* deleted were developed. Table 4 lists the primers used which were designed based on sequence information available in the NCBI database [GenBank accession No. AL009126.3 (*B. subtilis* 168) and CP012110.1 (*B. licheniformis* WX-02)].

**Table 4:** Primers used in this study

Primer name	Sequence 5′ → 3′^a^
Primers for *gdh* cloning	
*gdh*-F	CATG**CCATGG**GCATGTCAAAATCAGTAAAATC
*gdh*-R	CCG**CTCGAG**ATCGTGATAAGATTCTGC
Primers for *gdh* deletion	
Δ*gdh*-A-F	GC**TCTAGA**GATCTGACGACGAAAACAAG
Δ*gdh*-A-R	ACGGAAACGGCTTTTCGTCTAGGTAATTCCCCCTTCACTATC
Δ*gdh*-B-F	GATAGTGAAGGGGGAATTACCTAGACGAAAAGCCGTTTCCGT
Δ*gdh*-B-R	GC**GAGCTC**ATTATTATGGAGTACCGTGGATC
Δ*gdh*-F	TCTATCTGACCTTTGATAACGGCT
Δ*gdh*-R	GAATTTAGTGGTTCTGATCTGCATG
Primers for *acoR* deletion	
Δ*acoR*-A-F	GG**GGTACC**TGCCTCAGGCAGAGACATG
Δ*acoR*-A-R	CGTCATGCTTTGGGCGCCGAGAGGTCCATCCTCTCCTCATGCTTTTTC
Δ*acoR*-B-F	GAAAAAGCATGAGGAGAGGATGGACCTCTCGGCGCCCAAAGCATGACG
Δ*acoR*-B-R	CCG**CTCGAG**GGGTAAATGTGCTTCCTCCGCCT
Δ*acoR*-F	CGGAGTCCAATATGACAGG
Δ*acoR*-R	TCTGATATGAGCCATGACG
Primers for *gdh* complementation	
P43-*gdh*-TamyL-1	GC**TCTAGA**GCGGAATTTCCAATTTCATG
P43-*gdh*-TamyL-2	GATTTTACTGATTTTGACATGTGTACATTCCTCTCTTACC
P43-*gdh*-TamyL-3	GGTAAGAGAGGAATGTACACATGTCAAAATCAGTAAAATC
P43-*gdh*-TamyL-4	CGTCCTCTCTGCTCTTCTATCTTTTAATCGTGATAAGATTCTG
P43-*gdh*-TamyL-5	CAGAATCTTATCACGATTAAAAGATAGAAGAGCAGAGAGGACG
P43-*gdh*-TamyL-6	CGC**GGATCC**GATCACCCGCGATACCGTC

^a^Restriction sites highlight in bold. Underline stands for the overlap region for splicing by overlapping extension PCR (SOE-PCR)

To prepare the *gdh*-knock-out mutant, in-frame deletion of *gdh* gene in the genome of WX-02 was performed based on the method described previously [13]. Briefly, the 5′- and 3′-flanking sequences were amplified from the WX-02 genome using the primer pairs Δ*gdh*-A-F/Δ*gdh*-A-R and Δ*gdh*-B-F/Δ*gdh*-B-R, respectively. The two fragments were ligated by spliced overlap extension PCR (SOE-PCR) with the primers Δ*gdh*-A-F and Δ*gdh*-B-R. The fused fragment (1140 bp) was digested with *Xba*I and *Sac*I, and inserted into T2(2)-ori, generating plasmid T2Δ*gdh*. Transformation of *B. licheniformis* WX-02 with the knock-out plasmid T2Δ*gdh* was performed via electroporation based on the previous protocols [33]. The kanamycin-resistant transformants were picked and verified by PCR with the primers T2-VF and T2-VR. A positive clone was incubated in LB medium containing 20-μg/mL kanamycin at 45 °C for 8 h, then streaked onto LB agar with kanamycin for another 8-h incubation to obtain single-crossover recombinants. The recombinants were grown in LB medium at 37 °C with serial subcultures to promote homologous recombination. The kanamycin sensitive colonies resulting from a double crossover event were confirmed by PCR with the primers Δ*gdh*-F and Δ*gdh*-R (Additional file 3: Figure S3). The mutant strain was designated as WX-02Δ*gdh*. The *acoR* deletion mutant (WX-02Δ*acoR*) and *gdh*-*acoR* double-deletion mutant (WX-02Δ*gdh*Δ*acoR*) were prepared using the similar procedures described above. The two mutants were confirmed through PCR with the primer pairs Δ*acoR*-F/R and Δ*gdh*-F/R and Δ*acoR*-F/R, respectively (Additional file 3: Figure S3).

### Construction of the gene complementation strain of *B. licheniformis* WX-02Δ*gdh*

The P43 promoter was PCR-amplified from chromosomal DNA of *B. subtilis* 168 with the primers P43-*gdh*-TamyL-1 and 2 (Table 4). The *gdh* gene and terminator of the *amyL* gene were amplified from WX-02 genomic DNA with the primers P43-*gdh*-TamyL-3 to 6. The three fragments were fused by SOE-PCR with the primers P43-*gdh*-TamyL-1 and 6 (Table 4), and cloned into the *Xba*I and *Bam*HI sites of pHY300PLK, forming the plasmid pHY-*gdh.* The expression vector pHY-*gdh* was then transformed into *B. licheniformis* WX-02Δ*gdh* via electroporation based on a previously described method [34]. Positive transformants with tetracycline resistance were verified by PCR with the primers P43-*gdh*-TamyL-1 and 6 (Additional file 4: Figure S4), and was designated as WX-02Δ*gdh*/pHY-*gdh.*

### Cloning and expression of *gdh* gene in *E. coli* BL21(DE3)

The *gdh* gene was amplified from the genome of *B. licheniformis* WX-02 with the primers *gdh*-F and *gdh*-R (Table 4). The amplified product was ligated into the vector pET-28a(+) at *Nco*I and *Xho*I sites, resulting in the recombinant plasmid designated as pET-*gdh.* The plasmid was transformed into *E. coli* BL21(DE3) resulting in the recombinant strain *E. coli* BL21(DE3)/pET-*gdh* (Additional file 5: Figure S5) for protein expression. *E. coli* BL21(DE3)/pET-*gdh* was cultured in LB medium containing 20-μg/mL kanamycin at 37 °C to reach cell density (OD_600_) of 1.0. IPTG was then added to the culture to a final concentration of 0.2 mM. Cells were grown for another 6 h and harvested by centrifugation at 8000 *g* for 15 min (4 °C). The cell pellets were disrupted with an Ultra-high Pressure Continuous Flow Cell Disrupter at 1000–1500 bar and 4 °C. The cell lysate was centrifuged at 12,000 *g* for 30 min (4 °C) to remove residual cell debris. The enzyme in the supernatant was purified using a Ni–NTA purification kits. The purity and identity of the protein were determined by sodium dodecyl sulfate polyacrylamide gel electrophoresis (SDS-PAGE).

### Assay of activity and stereospecificity of glycerol dehydrogenase (GDH)

The activity of glycerol dehydrogenase (GDH) was assayed by measuring the change in absorbance at 340 nm corresponding to the oxidation of NADH or reduction of NAD^+^ at 37 °C. The assay was performed in Z buffer (pH 7.0) containing 60 mM Na_2_HPO_4_, 40 mM NaH_2_PO_4_, 10 mM KCl, 1 mM MgSO_4_, and 10 mM DTT [12]. We used either 4-mM NAD^+^ or 0.2-mM NADH as a coenzyme, dependent on the substrate used. Based on the enzyme (BDH or GDH) to be measured, substrates were glycerol, *D*-AC, *D*-2,3-BD and *meso*-2,3-BD, at a concentration of 50 mM. The reactions with different substrates were as follows:

$$D - {\text{AC }} + {\text{ NADH}}\overset {\text{BDH}} \longleftrightarrow 2,3 {\text{-BD }} + {\text{ NAD}}^{ + } ;\,{\text{Glycerol }} + {\text{ NAD}}^{ + } \overset {\text{GDH}} \longleftrightarrow {\text{DHA }} + {\text{ NADH}}$$

Here, one unit of activity was defined as the amount of enzyme required to consume or produce 1 μmol of NADH per minute. The specific activity of GDH was defined as the enzyme unit (U) divided by the amount of enzyme protein (mg). Protein concentrations were determined by the Bradford method [35].

### Analyses of metabolites during fermentation

Cells were harvested from the fermentation broth via centrifugation at 12,000*g* for 10 min. The cell free media was kept for analysis. The cell pellets were washed twice with deionized water and re-suspended with deionized water for determining cell density at 600 nm (OD_600_). Residual glucose concentration was measured enzymatically by a Bio-analyzer (SBA 40C, Shandong Academy of Sciences, China). Acetic acid, lactic acid, and ethanol were analyzed using an Agilent 1260 HPLC system. Analyte separation was achieved using Zorbax SB-Aq (4.6 mm ID × 25 0 mm, 5 μm) column with 99 % 20 mM Na_2_HPO_4_ and 1 % acetonitrile as the mobile phase (pH 2.0) at a flow rate of 0.5 mL/min. The column and detector temperatures were set at 35 °C; the injection volume was 10 μL and the detection wavelength was 210 nm. *D*-2,3-BD, *meso*-2,3-BD, and *D*-AC were analyzed by gas chromatography (Agilent Technologies 7890A) using the method described previously [13].

## Additional file

10.1186/s13068-016-0522-1 Multiple sequence alignments of GDH from *B. licheniformis* WX-02 (WX-02 GDH) with GDHs from other strains.
10.1186/s13068-016-0522-1 Phylogenetic analysis of amino acid sequences of GDHs and D-BDHs from different strains.
10.1186/s13068-016-0522-1 Confirmation of the recombinant strains of* B. licheniformis* by PCR amplification.
10.1186/s13068-016-0522-1 Confirmation of the mutant *B. licheniformis* WX-02Δ*gdh*/pHY-*gdh* strain by PCR amplification.
10.1186/s13068-016-0522-1 Analysis of the recombinant plasmid pET-*gdh* (A), and confirmation of the recombinant strain* E. coli *BL21(DE3)/ pET-*gdh* by PCR amplification (B).

## Abbreviations

BD: butanediol
AC: acetoin
BDH: 2,3-butanediol dehydrogenase
Gdh: glycerol dehydrogenase
AoDH ES: acetoin dehydrogenase enzyme system
AmyL: amylase
bp: base pairs
OD: optical density
SOE-PCR: splicing with overlapping extension PCR
DO: dissolved oxygen
