# Supplementary material for: Engineering Bacillus licheniformis for the production of meso-2,3-butanediol
Source: Biotechnol Biofuels. 2016 Jun 2;9:117. doi: 10.1186/s13068-016-0522-1 (PMC4890260; doi:10.1186/s13068-016-0522-1)
Supplement: Supplementary file 1 — 10.1186/s13068-016-0522-1 Multiple sequence alignments of GDH from B. licheniformis WX-02 (WX-02 GDH) with GDHs from other strains. [file 13068_2016_522_MOESM1_ESM.pdf]

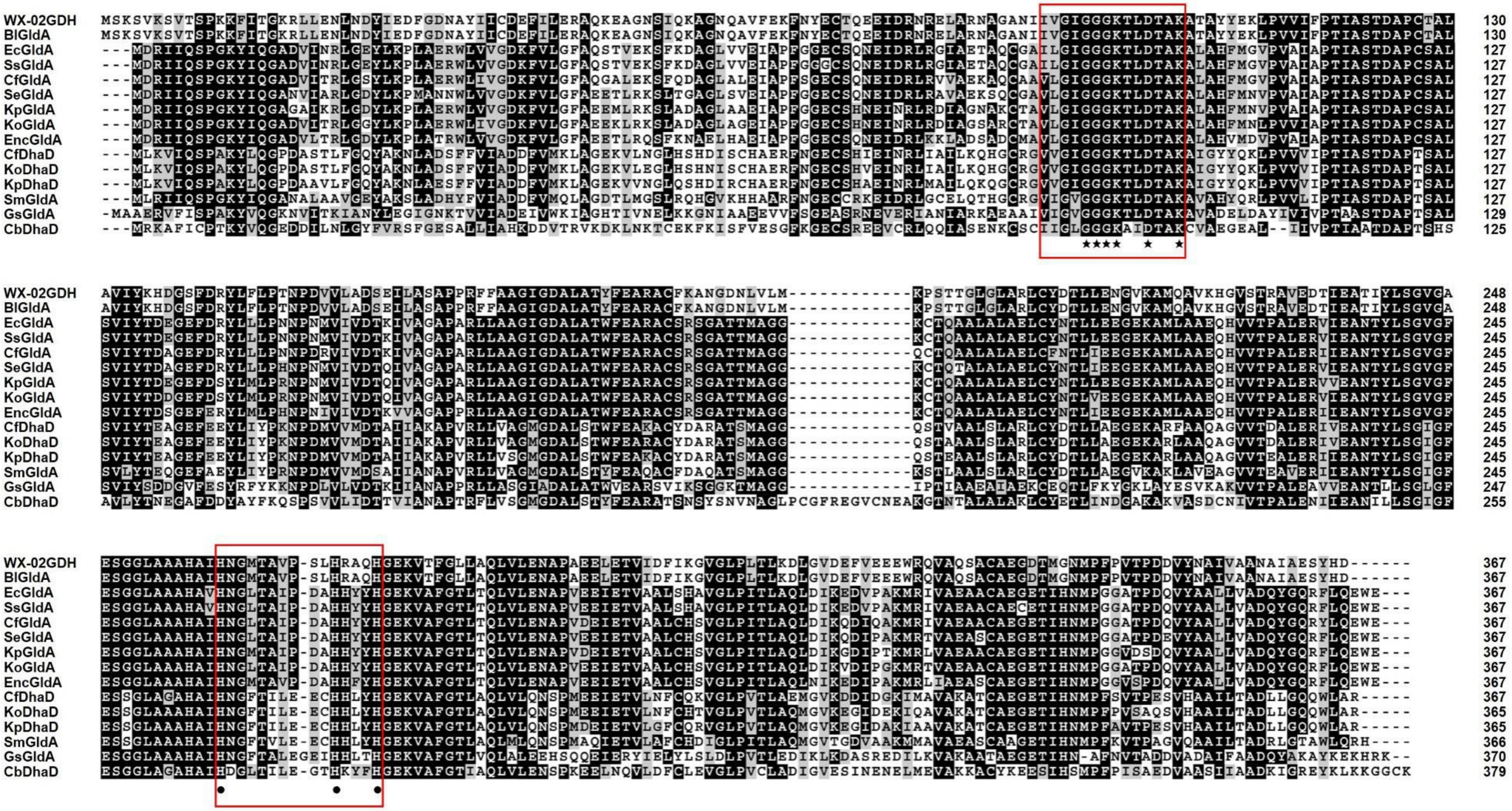
WX-02GDH
BlGldA
EcGldA
SsGldA
CfGldA
SeGldA
KpGldA
KoGldA
EncGldA
CfDhaD
KoDhaD
KpDhaD
SmGldA
GsGldA
CbDhaD

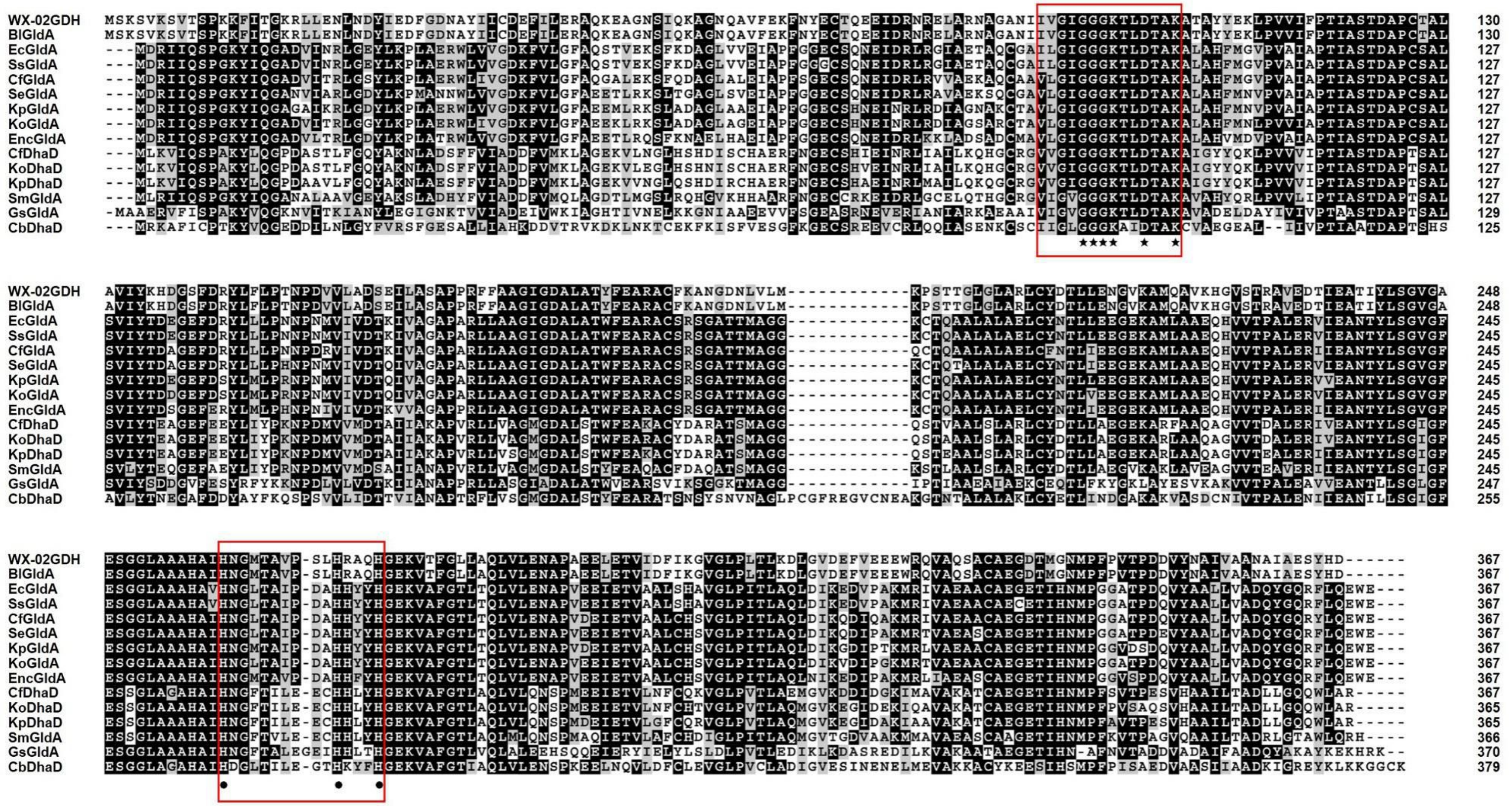

Figure S1. Multiple sequence alignments of GDH from *B. licheniformis* WX-02 (WX-02 GDH) with GDHs from other strains [*B. licheniformis* 10-1-A (BlGldA, AHW84748.1), *Escherichia coli* (EcGldA, P0A9S6), *Shigella sonnei* (SsGldA, YP_005459262.1), *Citrobacter freundii* (CfGldA, WP_003847842.1), *Salmonella enterica* (SeGldA, YP_006888481.1), *Klebsiella pneumoniae* 280_1220 (KpGldA, EPP12735.1), *Klebsiella oxytoca* (KoGldA, YP_005017437.1), *Enterobacter cloacae* (EncGldA, AFM62175), *Citrobacter freundii* (CfDhaD, P45511.1), *Klebsiella oxytoca* (KoDhaD, YP_005016612), *Klebsiella pneumoniae* CGMCC1.6366 (KpDhaD,

KJ206474.1), *Serratia marcescens* H30 (SmGldA) [1], *Geobacillus stearothermophilus* (GsGldA, P32816), *Clostridium butyricum* (CbDhaD, AAN17729)]. The boxes indicate the putative coenzyme binding motif proposed as the signature of the iron-dependent ADH, and the box below indicates the residues involved in metal binding. The location of the conserved residues corresponding to coenzyme and metal binding sites are also marked with star and dot, respectively. Amino acid residues are numbered on the right.
