# Supplementary material for: Engineering Bacillus licheniformis for the production of meso-2,3-butanediol
Source: Biotechnol Biofuels. 2016 Jun 2;9:117. doi: 10.1186/s13068-016-0522-1 (PMC4890260; doi:10.1186/s13068-016-0522-1)
Supplement: Supplementary file 2 — 10.1186/s13068-016-0522-1 Phylogenetic analysis of amino acid sequences of GDHs and D-BDHs from different strains. [file 13068_2016_522_MOESM2_ESM.pdf]

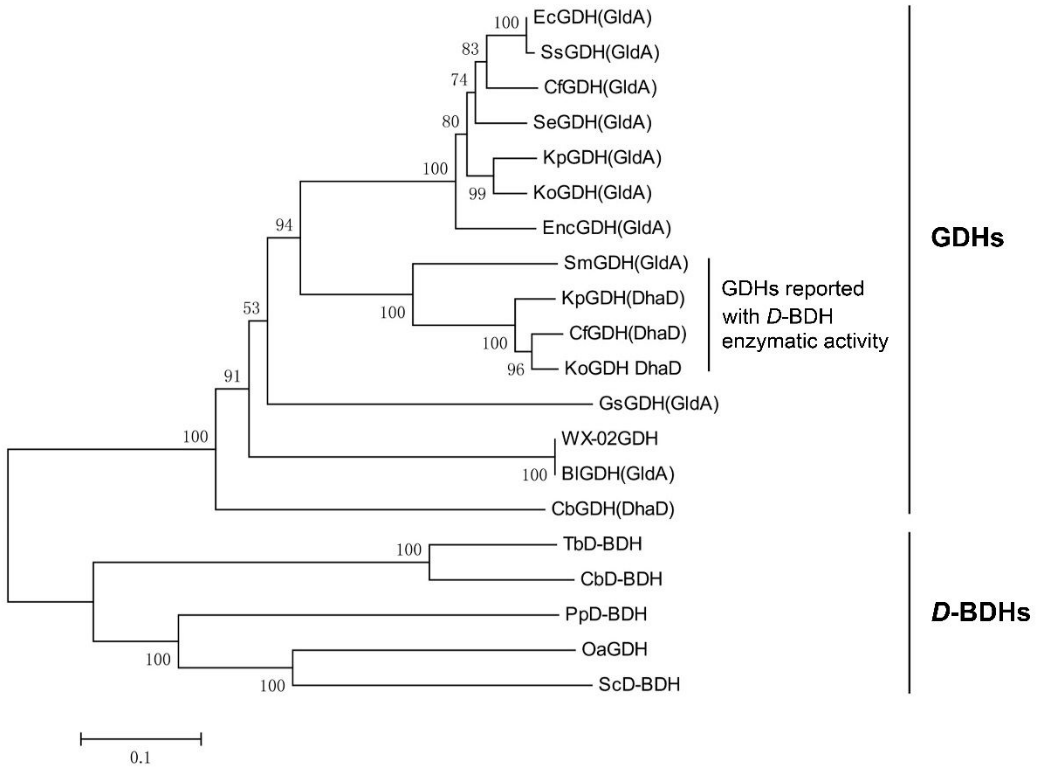

Figure S2. Phylogenetic analysis of amino acid sequences of GDHs and *D*-BDHs from different strains. The phylogenetic tree is constructed with the neighbor-joining method (1000 bootstraps) using the MEGA 5.1 program [29]. The sequences compared include GDHs mentioned in Fig. S4 and *D*-BDHs from *Thermoanaerobacter brockii* (TbD-BDH, CAA46053), *Clostridium beijerinckii* (CbD-BDH, AAA23199), *Paenibacillus polymyxa* (PpD-BDH, AEV53933.1), *Saccharomyces cerevisiae* (ScD-BDH NP_009341) and GDH from *Ogataea angusta* (OaGDH, BAD32688).
