# Supplementary material for: Engineering Bacillus licheniformis for the production of meso-2,3-butanediol
Source: Biotechnol Biofuels. 2016 Jun 2;9:117. doi: 10.1186/s13068-016-0522-1 (PMC4890260; doi:10.1186/s13068-016-0522-1)
Supplement: Supplementary file 3 — 10.1186/s13068-016-0522-1 Confirmation of the recombinant strains of B. licheniformis by PCR amplification. [file 13068_2016_522_MOESM3_ESM.pdf]

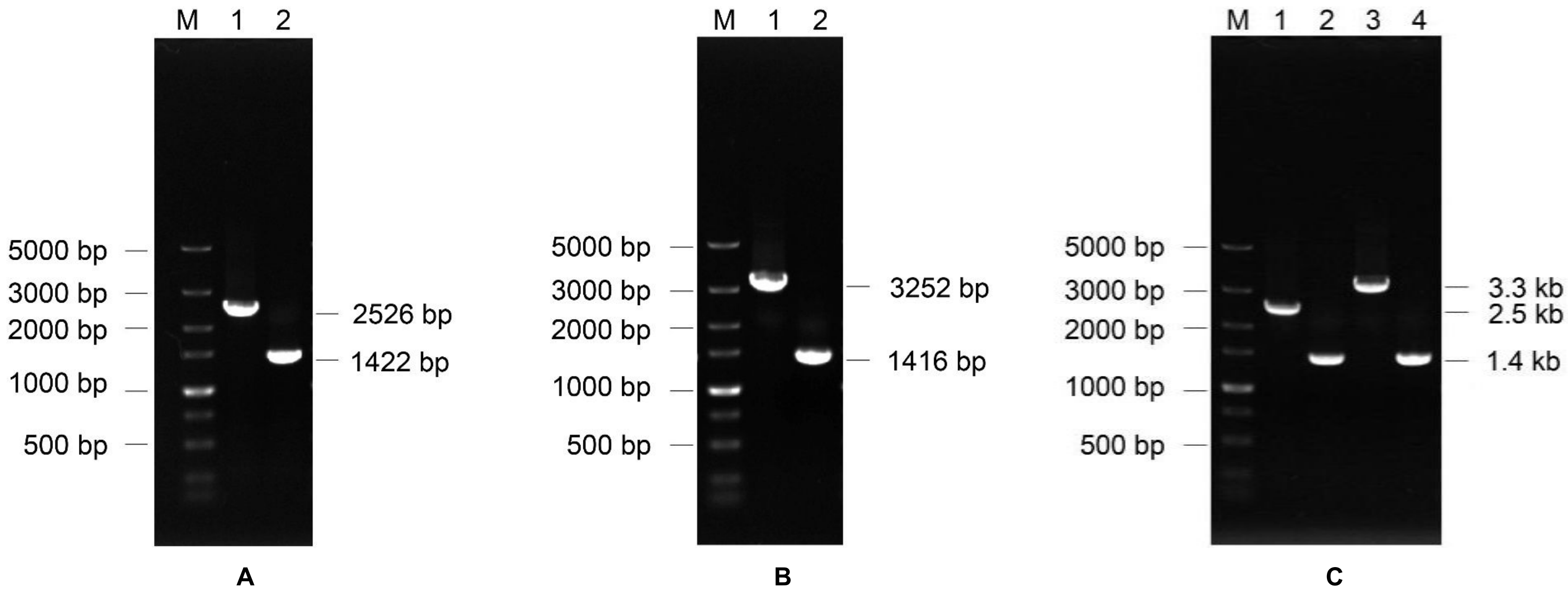

Figure S3. Confirmation of the recombinant strains of *B. licheniformis* by PCR amplification.

(A) *Lane M*: DL5000 marker; *Lane 1*: negative control (PCR products amplified from the wild strain WX-02 genome); *Lane 2*: PCR products amplified from WX-02Δ*gdh* with the primers Δ*gdh*-F and Δ*gdh*-R

(B) *Lane M*: DL5000 marker; *Lane 1*: negative control (PCR products amplified from the wild strain WX-02 genome); *Lane 2*: PCR products amplified from WX-02Δ*acoR* with the primers Δ*acoR*-F and Δ*acoR*-R

(C) *Lane M*: DL5000 marker; *Lane 1 and 3*: negative control (PCR products amplified from the wild strain WX-02 genome) with the primer pairs Δ*gdh*-F/R and Δ*acoR*-F/R, respectively ; *Lane 2 and 4*: PCR products amplified from WX-02Δ*gdh*Δ*acoR* with the primer pairs Δ*gdh*-F/R and Δ*acoR*-F/R, respectively .
