# Supplementary material for: Engineering Bacillus licheniformis for the production of meso-2,3-butanediol
Source: Biotechnol Biofuels. 2016 Jun 2;9:117. doi: 10.1186/s13068-016-0522-1 (PMC4890260; doi:10.1186/s13068-016-0522-1)
Supplement: Supplementary file 4 — 10.1186/s13068-016-0522-1 Confirmation of the mutant B. licheniformis WX-02Δgdh/pHY-gdh strain by PCR amplification. [file 13068_2016_522_MOESM4_ESM.pdf]

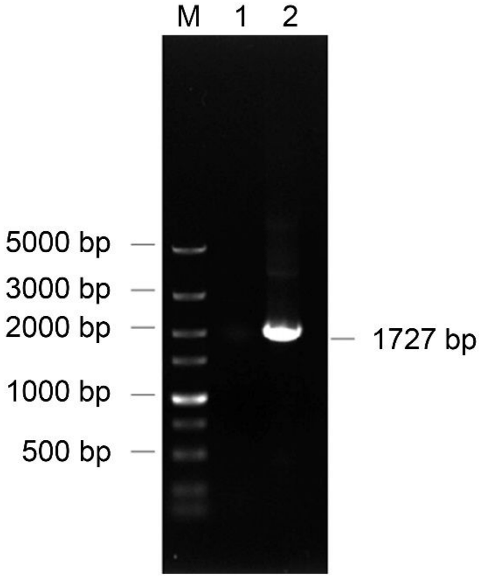

Figure S4. Confirmation of the mutant *B. licheniformis* WX-02Δ*gdh*/pHY-*gdh* strain by PCR amplification.

Lane M: DL5000 marker; *Lane 1*: negative control (PCR products amplified from the wild strain WX-02); *Lane 2*: PCR products of fusion fragment of P43-*gdh*-TamyL from WX-02Δ*gdh*/pHY-*gdh* with the primers P43-*gdh*-TamyL-1 and 6.
