# Supplementary material for: Engineering Bacillus licheniformis for the production of meso-2,3-butanediol
Source: Biotechnol Biofuels. 2016 Jun 2;9:117. doi: 10.1186/s13068-016-0522-1 (PMC4890260; doi:10.1186/s13068-016-0522-1)
Supplement: Supplementary file 5 — 10.1186/s13068-016-0522-1 Analysis of the recombinant plasmid pET-gdh (A), and confirmation of the recombinant strain E. coli BL21(DE3)/ pET-gdh by PCR amplification (B). [file 13068_2016_522_MOESM5_ESM.pdf]

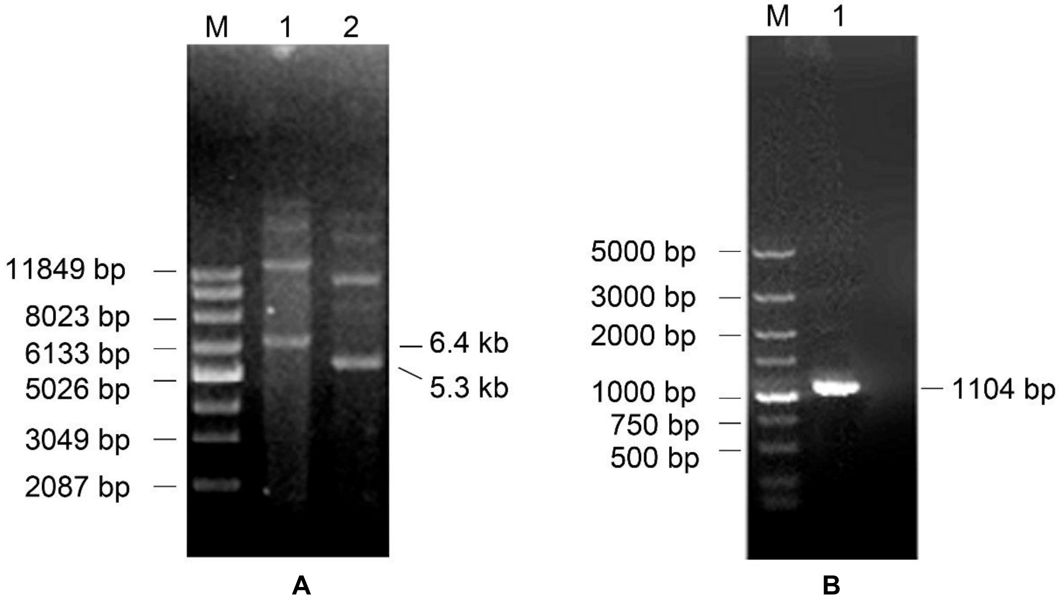

Figure S5. Analysis of the recombinant plasmid pET-*gdh* (**A**), and confirmation of the recombinant strain *E. coli* BL21(DE3)/ pET-*gdh* by PCR amplification (**B**)

(A) *Lane M*: Supercoiled DNA Ladder Marker; *Lane 1*: plasmid pET-*gdh* (6.4 kb); *Lane 2*: plasmid pET-28a(+) (5.3 kb)

(B) *Lane M*: DL5000 Marker; *Lane 1*: colony PCR products of the transformant BL21(DE3)/ pET-*gdh* (1.1 kp)
